# Histopathological alterations in airways associated with physiological changes in airway allergy phenotypes

**DOI:** 10.3389/falgy.2026.1771120

**Published:** 2026-02-26

**Authors:** Marisol Alvarez-González, Angélica Flores-Flores, Ivonne Pacheco-Alba, Blanca Bazán-Perkins

**Affiliations:** 1 Laboratorio de Inmunofarmacología, Instituto Nacional de Enfermedades Respiratorias Ismael Cosio Villegas, Mexico City, Mexico; 2 Tecnologico de Monterrey, Escuela de Medicina y Ciencias de la Salud, Mexico City, Mexico

**Keywords:** airway hyperresponsiveness, allergy, asthma model, guinea pig, integrin β1 subunit, non-responders

## Abstract

**Introduction:**

Ovalbumin sensitization in guinea pigs induces diverse allergic responses. The asthma model exhibits airway obstruction, hyperresponsiveness, fibrosis, and reduced airway caliber, associated with elevated β1 integrin subunit expression. In contrast, the non-responder (NR) phenotype shows no obstruction or hyperresponsiveness under chronic antigen exposure. It is likely that NR guinea pigs lack increased β1 integrin subunit expression due to the absence of a typical asthma response. This study aimed to compare the histopathological and pathophysiological characteristics between the asthma model and NR phenotype in ovalbumin-sensitized guinea pigs to understand the differences in airway β1 integrin subunit expression.

**Methods:**

Guinea pigs were sensitized and challenged with ovalbumin nine times at 10-day intervals. The animals were then categorized into either the asthma model or the NR group. After the ninth antigen challenge, baseline obstruction, antigen-induced airway hyperresponsiveness, and immunohistopathological changes were evaluated.

**Results:**

Airway hyperresponsiveness to histamine was only observed in the asthma model. Both asthma and NR groups had increased basal obstruction and accumulation of the integrin β1 subunit in the subepithelial region compared to controls, with a greater increase in NR. Integrin β1 subunit expression in airway smooth muscle was higher in the asthma model than in NR. The subepithelial area was enlarged in both asthma and NR groups compared to controls. Basal caliber reduction was correlated with fibrosis and integrin β1 subunit in the subepithelial region.

**Discussion:**

Fibrosis and deposition of the β1 integrin subunit in the subepithelial region are associated with baseline obstruction but not with the magnitude of airway obstruction or hyperresponsiveness. In the asthma model, the airway smooth muscle phenotype, characterized by high β1 integrin subunit, could influence contraction and hyperreactivity.

## 1 Introduction

The baseline caliber of the airway is essential for maintaining pulmonary ventilation. It has been observed that the airways expand through smooth muscle relaxation during inhalation and contract during exhalation (1). This dynamic adjustment suggests that airway diameter is regulated by the spontaneous contraction–relaxation cycle, or tone changes, of the airway smooth muscle. However, the caliber shows significant changes during the development of airway diseases, where airway tone homeostasis is lost, increasing resistance to airflow and air trapping (2). This increase is associated with airway contractile capacity and airway hyperresponsiveness through the tonic activation of airway smooth muscle (3–5). Alterations in airway basal tone can also be attributed to airway wall enlargement due to structural modifications (6). Subepithelial fibrosis, which leads to extracellular matrix expansion, is frequently observed even in cases of childhood asthma and has been associated with disease severity (7). One proposed functional outcome of the thickening in the subepithelial region is the enhancement of airway smooth muscle contraction by reducing airway distensibility and facilitating smooth muscle adaptation to shorter lengths (8, 9).

Various phenotypes of allergic response to ovalbumin (OVA) can naturally occur in guinea pigs. These include all characteristics of the asthma model, such as airway obstruction and hyperresponsiveness following antigenic challenge. Others also fit the asthma model but with variable responses, sometimes showing obstruction after the challenge but not hyperreactivity. Another phenotype includes those that never obstruct after the challenge and do not show hyperreactivity in chronic models, known as non-responders (NR). This NR phenotype occurs naturally in approximately 20% of guinea pigs that are sensitized and challenged. NR guinea pigs, like the asthma models, are characterized by serum levels of anti-OVA IgE and IgG1, but are differentiated by high levels of IFN-γ and high expression of β2 laminin in smooth muscle (10–12).

A key characteristic of chronic asthma in humans and the guinea pig asthma model is the increase in baseline airway obstruction and subepithelial fibrosis (13, 14). In the guinea pig asthma model, there is an observed increase in the expression of the β1 integrin subunit in airway smooth muscle, which is associated with the magnitude of antigen-induced obstruction, airway hyperreactivity, and baseline obstruction (15). This is likely due to the fact that cytokines characteristic of allergic asthma, such as IL-13, can induce the activation of the β1 integrin subunit. Moreover, the blockade of integrin β1, such as α2β1, inhibits pathological force generation by airway smooth muscle by preventing adhesion to extracellular matrix proteins (16). In addition, another study confirmed the effect of IL-13 in increasing the activation of the β1 integrin subunit and observed that IL-17 also has a similar effect on β1 integrin activation, contributing to muscle hypercontractility (17). Given that NR guinea pigs do not exhibit the pathophysiological characteristics of the asthma model, it is likely that they do not present increased levels of β1 integrin subunit expression due to the absence of typical asthma features. To evaluate this hypothesis, in the present study, we compared the levels of β1 integrin subunit expression in the asthma model and the NR guinea pig phenotypes, examining their association with hyperreactivity, obstruction, airway tone, and the degree of subepithelial fibrosis between both models. 

## 2 Materials and methods

### 2.1 Animals

In general, both female and male guinea pigs can be used in allergic asthma models. However, during the development of our experimental model in both sexes, we observed considerable variability in the responses to the antigen. In females, young individuals (≈450 g) did not develop airway hyperresponsiveness, whereas adult females (>550 g) consistently did (unpublished data). This finding is consistent with previous reports by Regal et al. (18). To avoid this age-related variability in airway hyperresponsiveness, and considering that the model requires the use of young guinea pigs, only male guinea pigs were included in the present study. We utilized male outbred guinea pigs, each weighing 400 g, sourced from Harlan Mexico City, Mexico (strain HsdPoc:DH). The guinea pigs were housed in our institutional laboratory, which provided filtered air conditioning at 21°C ± 1°C with a humidity level of 50%–70%. They were exposed to a 12/12-h light/dark cycle and fed sterilized pellets (2040 Harlan Teklad Guinea Pig Diet, WI, USA) with unlimited access to water. All handling followed protocols approved by the Scientific and Bioethics Committee of the Instituto Nacional de Enfermedades Respiratorias (IRB organization information: IORG0003948).

### 2.2 Antigen challenges to identify asthma model and NR guinea pigs

Antigen challenges were used to identify asthma model and NR guinea pigs. The sensitization process involved intraperitoneal (0.5 mg/mL) and subdermal (0.5 mg/mL) administration of a combination of OVA (chicken egg albumin grade II; 60 μg/mL, Sigma, St. Louis, MO, USA) and 1 mg/mL aluminum hydroxide (J.T. Baker, Phillipsburg, NJ, USA) in physiological saline solution. Eight days later, sensitization was enhanced with a 5-min OVA aerosol exposure (3 mg/mL). Starting from day 15, the guinea pigs were challenged with OVA aerosol every 10 days: 1 mg/mL for the first challenge and 0.5 mg/mL for subsequent challenges. The control guinea pigs underwent nine challenges with saline solution. Aerosols were produced using a US-1 Bennett nebulizer, generating particles of various sizes: <4 µm (44%), 4–10 µm (38%), and >10 µm (18%). Inhalation challenges were conducted in a whole-body plethysmograph to evaluate pulmonary function (19). The Buxco Bio System XA software calculated respiratory parameters, recording the bronchoobstructive index (Bi) both basally and during OVA challenges (20). Obstructive response magnitudes classified guinea pigs into the asthma model (*n* = 6), which consistently showed obstruction, and “non-responders” (NR, *n* = 6), which showed no obstruction. Control animals (*n* = 6) underwent sham challenges with saline solution.

### 2.3 Antigen-induced airway hyperresponsiveness

Airway responsiveness was assessed after the twelfth OVA challenge by comparing histamine (Sigma, St. Louis, MO, USA) dose–response curves before and after OVA administration at the study's conclusion. Non-cumulative doses of histamine (0.001–0.32 mg/mL) were administered after acquiring a baseline Bi. Each dose was delivered over 1 min, and the average Bi value for the following 5 min was recorded. Doses were separated by 10-min intervals. Dose–response curves were halted when Bi reached three times the baseline level. After Bi returned to near-baseline levels (<50% increment) (21), an OVA challenge was administered, and the second curve was performed 3 h later.

### 2.4 Structural changes induced by antigenic challenges

One hour after completing the second histamine curve, the guinea pigs were euthanized with an intraperitoneal injection of pentobarbital sodium (65 mg/kg; Pfizer, Mexico). The left caudal lung lobe was dissected and fixed via manual perfusion with 10% neutral buffered formaldehyde solution through the intra-arterial route until fully exsanguinated. Lung fragments were embedded in paraffin, sectioned at 4 µm thickness, stained with Masson trichrome, and analyzed using automated morphometry to measure the surface areas of the subepithelial (lamina propria) and smooth muscle regions. Measurements were taken from six randomly selected bronchioles per animal, adjusted by the corresponding basement membrane length, and averaged to obtain final results. Differentiation between bronchi and bronchioles was based on the presence or absence of cartilage in the airway wall.

### 2.5 β1-integrin expression induced by antigenic challenges

The paraffin-embedded lung tissues, originally used for morphometry, were also utilized in immunohistochemistry. Sections, 3 µm thick, underwent deparaffinization at 55°C for 30 min and were subsequently rehydrated through a series of graded alcohols and distilled water. Antigen retrieval was achieved using a 10 mm citrate buffer (pH 6). To inhibit endogenous peroxidase, samples were treated with 3% hydrogen peroxide. Blocking of non-specific binding sites was done with 2% horse serum. Sections were incubated overnight at 4°C with a primary antibody against β1-integrin (1:75 dilution, clone P4G11 mouse monoclonal IgG1; Chemicon Int., Temecula, CA, USA). Specific binding was detected using the R.T.U. Vectastain Universal Quick Kit (Vector Laboratories, Inc., Burlingame, CA, USA), following the sequential incubation steps of blocking serum, secondary antibody, and streptavidin/peroxidase complex. The chromogen used was 3-amino-9-ethylcarbazole (BioGenex, Fremont, CA, USA), and the sections were counterstained with Mayer's hematoxylin. Throughout the procedure, rinsing with 0.1% phosphate-buffered saline and Tween 20 was performed. For controls against non-specific binding, sections without primary antibodies showed no positive staining, and rabbit IgG (Southernbiotech, Birmingham, AL, USA) isotype controls were also negative. β1-integrin immunostaining areas were quantified using an automated image analyzer (Qwin, Leica Microsystems, Wetzlar, Germany) by evaluating positive and negative areas across five random subepithelial and smooth muscle fields at 200× magnification.

### 2.6 Statistical analysis

Airway responsiveness to histamine was evaluated using the provocative dose 200% (PD_200_)—defined as the interpolated dose causing a 3-fold increase in basal Bi. For multiple comparisons, two-way ANOVA followed by Tukey's test was applied. Associations were assessed through Spearman correlation coefficients, with statistical significance determined according to the Bonferroni correction.

## 3 Results

### 3.1 Selection of asthma model and NR guinea pigs

**Figure 1:**
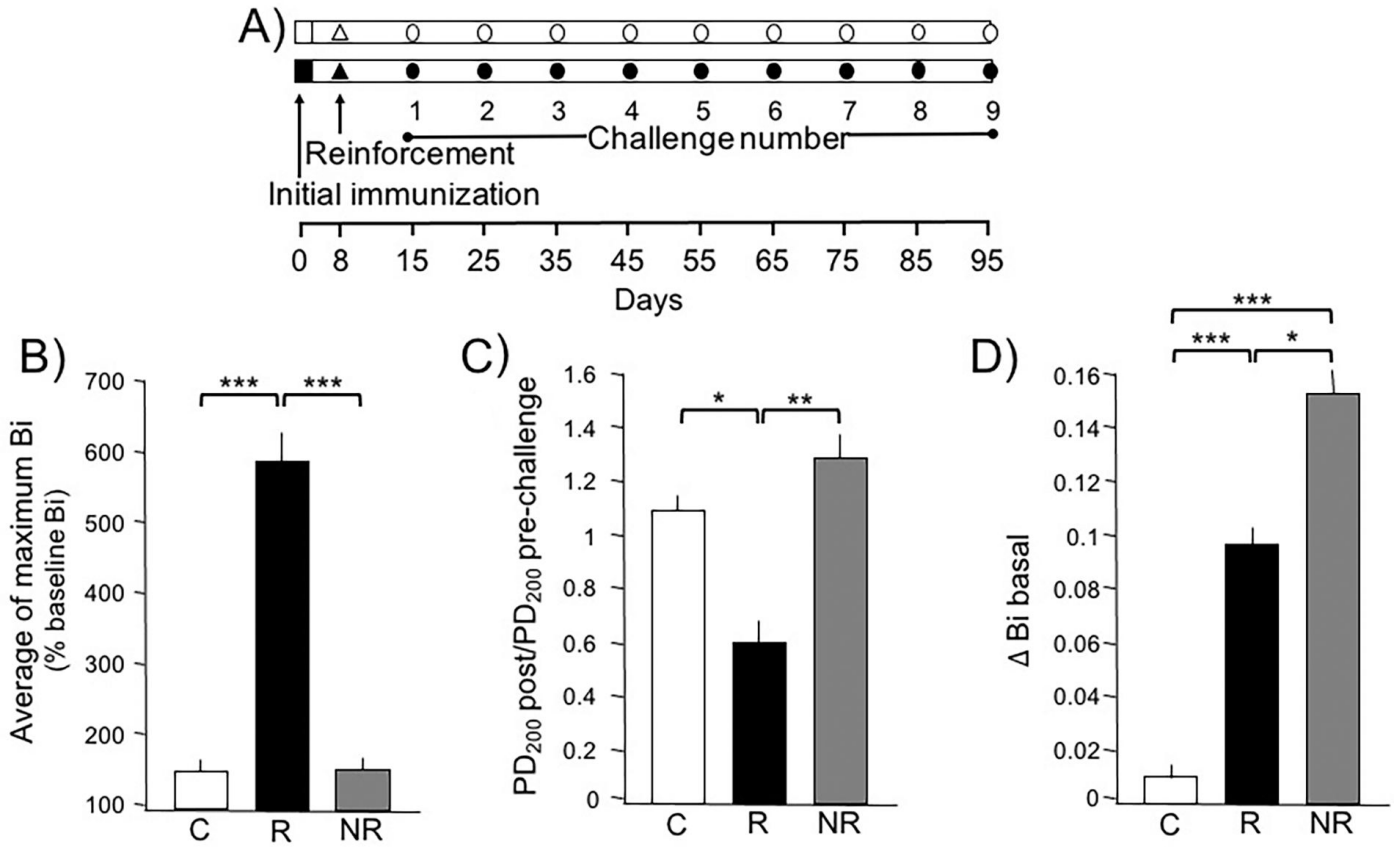
Changes in basal bronchoconstriction index (Δ Bi basal) in asthma model (responder, R) and non-responder (NR) phenotypes to antigenic challenge. **(A)** Experimental design. The empty symbols represent guinea pigs that were challenged and sensitized with physiological saline solution, while the filled symbols represent those that received the antigen. **(B)** During each challenge, the Bi was assessed and the average of all the maximum Bi reached in each guinea pig is shown for the group where Bi always increased after the antigenic challenge (R group), those that never increased Bi after the challenge (NR group), and the controls **(C)** challenged with saline. **(C)** Antigen-induced airway responsiveness to histamine in guinea pigs in the last (ninth) challenge with the antigen. Airway responsiveness to histamine was evaluated by the provocative dose 200% (PD_200_), i.e., the interpolated histamine dose that caused a 3-fold increase in the basal Bi before and after the antigenic challenge. **(D)** Δ Bi basal in the first challenge compared to the basal Bi value in the last antigenic challenge in the R and NR guinea pigs or with saline in the controls. *p < 0.05, **p < 0.01, and ***p < 0.001, comparison between groups, Tukey's *post hoc* test. Bars represent the means ± SD of *n* = 6 animals of each group.

Guinea pigs can naturally exhibit or fail to exhibit a bronchoobstructive response to antigen challenges. To distinguish asthma model and NR phenotypes, we evaluated the maximum Bi values reached after nine antigen challenges in OVA-sensitized guinea pigs. We selected six guinea pigs for the asthma model group that consistently showed a transient obstructive response to each antigenic challenge, with Bi values consistently ≥200% of the basal Bi. We then selected six guinea pigs that showed no response at all for the NR group. The average maximum Bi response in the asthma model group was significantly higher than in both the control and NR groups (*p* < 0.001 each; Figure 1B). The control group, which was challenged with saline, exhibited Bi values similar to those of the NR group.

### 3.2 Antigen-induced airway hyperresponsiveness

The PD_200_ values to aerosolized histamine in both NR and control guinea pigs were not significantly different before and after the OVA challenge. However, guinea pigs in the asthma model group exhibited a significant decrease in histamine PD_200_ after the OVA challenge compared to their basal PD_200_ values. The ratio of PD_200_ after the antigenic challenge to PD_200_ before the challenge was significantly lower in the asthma model group compared to the control and NR groups (*p* < 0.045 and *p* < 0.0052, respectively; Figure 1C).

### 3.3 Antigen-induced changes in baseline Bi

Baseline Bi values remained similar in control animals throughout the study. In contrast, OVA-challenged guinea pigs showed a progressive increase in baseline Bi values. The change from the first to the last baseline Bi values is shown in Figure 1D as the Δ Bi basal. Compared to the Δ Bi basal control group, the asthma model and NR values were significantly higher (*p* < 0.001 each). The Δ Bi basal in the NR group was higher than that observed in the asthma model group (*p* < 0.0134).

### 3.4 Airway structural changes induced by antigen challenges

**Figure 2:**
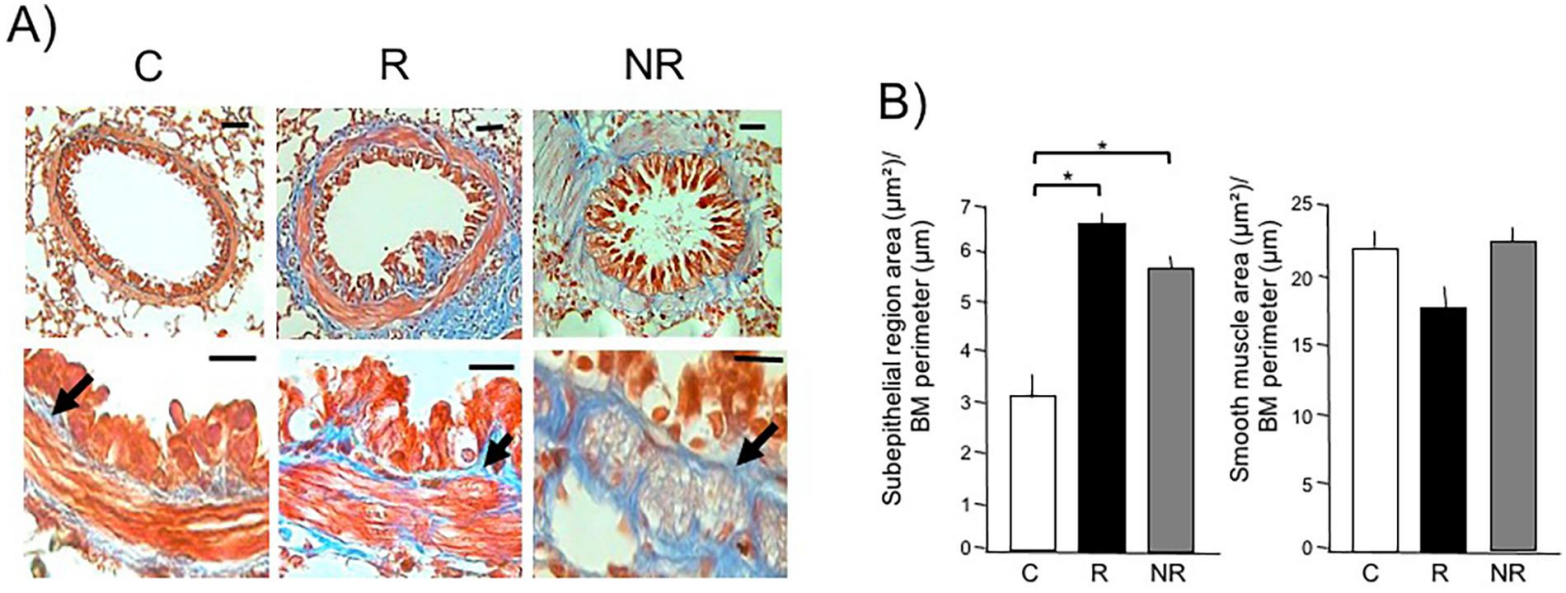
Distinctive histological areas of the airway subepithelial region and smooth muscle in asthma model (responder, R) and non-responder (NR) phenotypes to antigenic challenge. **(A)** Micrographs from the lungs of control (C), R, and NR guinea pigs show a representative bronchiole with subepithelial (arrows, blue stain) and smooth muscle (red stain) regions. The top images are magnified 10× with a scale bar representing 30 μm; the bottom images are magnified 40× with a scale bar representing 15 μm. **(B)** Graphs showing the areas of the subepithelial and smooth muscle regions of the bronchiole, adjusted by the basement membrane (BM) perimeter, measured using automated morphometry. Bars and vertical lines represent the mean ± SD of *n* = 6 animals in each experimental group. *p < 0.001; one-way ANOVA with Tukey’s *post hoc* test.

Masson trichrome staining revealed collagen deposits as continuous blue bands with a clear distinction of smooth muscle layers in bronchioles. Automated morphometric analysis demonstrated a statistically significant increase in the subepithelial region in bronchioles in both the asthma model and NR groups compared to controls (*p* < 0.001 each). No changes were observed in the magnitude of smooth muscle layers among groups (Figure 2).

### 3.5 β1-integrin subunit expression changes induced by antigen

**Figure 3:**
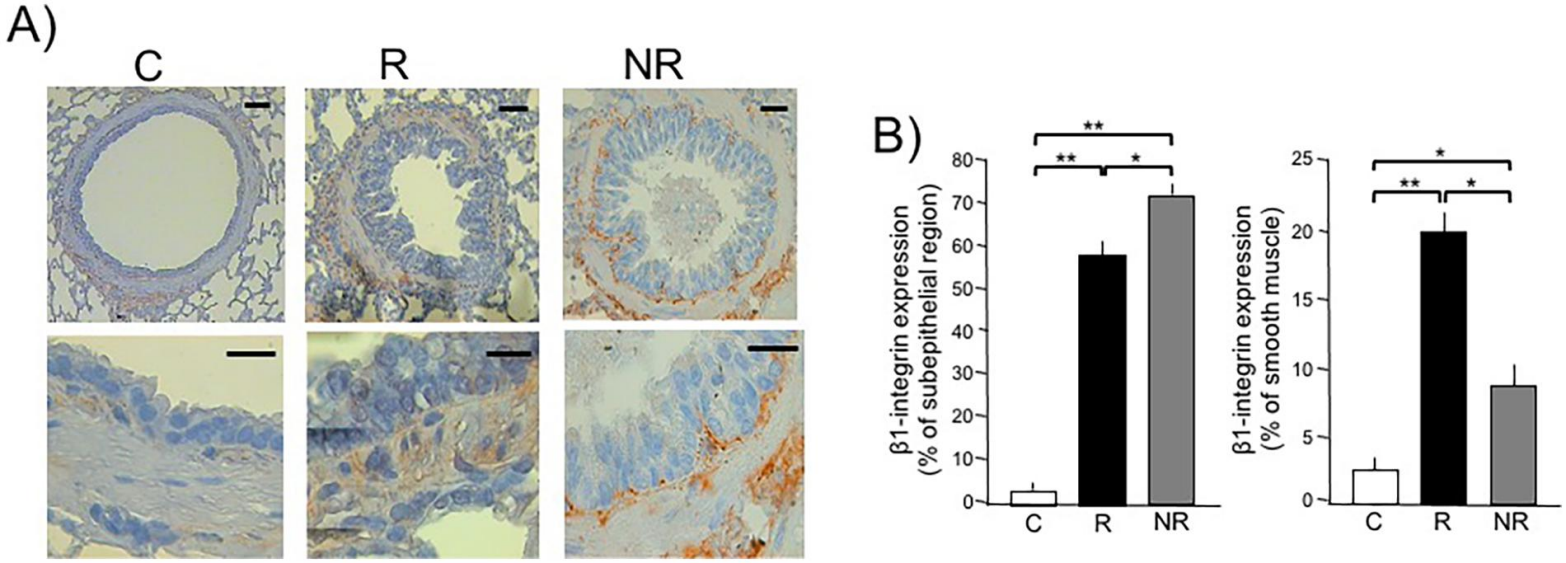
Determination of β1-integrin expression in airway subepithelial region and smooth muscle in asthma model (responder, R) and non-responder (NR) phenotypes to antigenic challenge in guinea pigs by immunohistochemistry. **(A)** Representative micrographs from control (C) and R and NR guinea pig showing the immunostaining to β1-integrin in subepithelial and muscle cells regions. The top images are magnified 10× with a scale bar representing 30 μm; the bottom images are magnified 40× with a scale bar representing 15 μm. **(B)** Graphs showing the area of β1-integrin subunit expression, using semiquantitative analysis of β1-integrin immunostaining using 3-amino-9-ethylcarbazole as chromogen. The positive staining area was quantified by automated morphometric analysis using an automated image analyzer (Qwin, Leica Microsystems, Wetzlar, Germany). Regions of interest were defined as the subepithelial region and airway smooth muscle bundles. For each animal, five non-overlapping fields per section were analyzed at 40× magnification. The area of positive staining was expressed as μm^2^ (or percentage of total area) and averaged per animal. Bars and vertical lines represent the mean ± SD of *n* = 6 animals in each experimental group. **p < 0.01 and *p < 0.01; one-way ANOVA with Tukey’s *post hoc* test.

**Figure 4:**
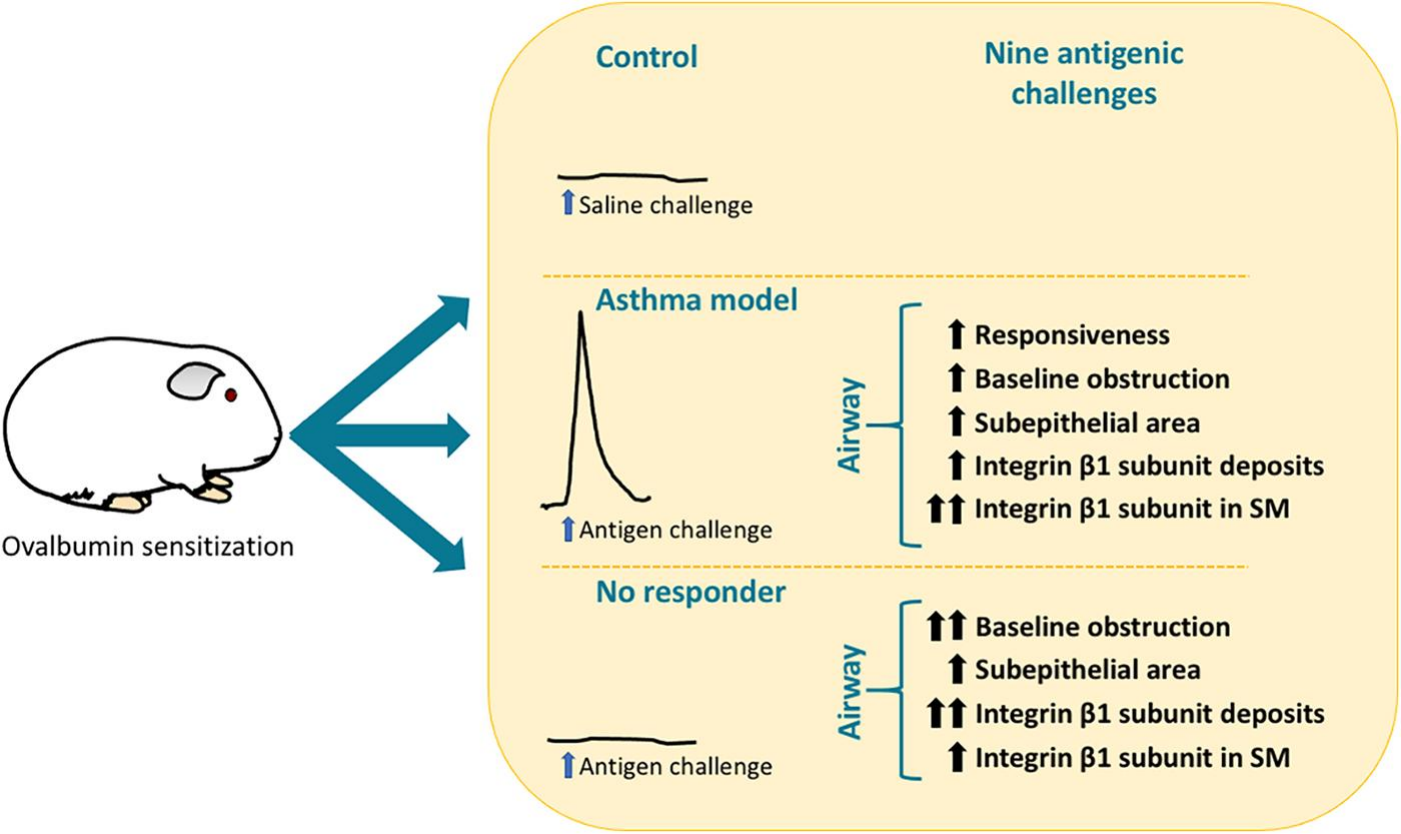
Summary of the main findings in guinea pigs from the asthma model and the non-responder phenotype following antigenic challenge. SM, smooth muscle.

In the ninth OVA-challenged group, the β1-integrin subunit showed overexpression in the subepithelial region in bronchioles of both the asthma model and NR groups compared to controls (Figure 3; *p* < 0.001). The expression of the β1-integrin subunit in the subepithelial region in the NR group was significantly higher than that in the asthma model group (*p* < 0.0014). The expression levels of the β1-integrin subunit in bronchiole smooth muscle increased significantly in the asthma model group compared to the control and NR groups (*p* < 0.001 and *p* < 0.0019, respectively), while the NR group showed higher β1-integrin subunit expression than controls(*p* < 0.002; Figure 3). A summary of the main findings in guinea pigs from the asthma model and the non-responder phenotype following antigenic challenge is presented in Figure 4.

### 3.6 Relationship between Δ Bi basal and structural changes in bronchioles

The relationship between the Δ Bi basal and structural changes in the bronchioles was investigated. The extent of the bronchiole subepithelial region correlated with the magnitude of Δ Bi basal (*p* < 0.001), but no relationship was observed between Δ Bi basal and the mass of bronchiolar smooth muscle (Table 1). The magnitude of Δ Bi basal was associated with the presence of β1-integrin deposited in the subepithelial area (*p* < 0.001). There was no relationship between the ratio of PD_200_ after antigen challenge to PD_200_ before the challenge and either the maximum Bi value reached after the last antigen challenge or basal Bi (*r* = 0.168, *P* = 0.25 and *r* = −0.22, *P* = 0.14, respectively).

**Table 1:** Spearman correlation (*r*_*s*_) between the changes in baseline bronchoobstructive index (Δ Bi) and selected variables.

Parameter	Airway region/Compartment	Δ Bi baseline
Area (µm^2^)/basement membrane perimeter (µm)	Subepithelial region	*r*_*s*_ = **0.714**, *p* = 0.0004*
	Smooth muscle	*r*_*s*_ = **0.066**, *p* = *0.39*
β1-integrin expression	Subepithelial region	*r*_*s*_ = **0.833**, *p* = 0.000009*
	Smooth muscle	*r*_*s*_ = **0.497**, *p* = *0.017*

* Statistically significant values at the adjusted level using the Bonferroni correction (*p* < 0.0125).

## 4 Discussion

The phenotypes of allergic responses in guinea pig models of asthma and chronic non-responsive (NR) airways are clearly distinct. The asthma model is characterized by antigen-induced airway obstruction and hyperreactivity, whereas the chronic NR model does not exhibit these responses.

In humans, increased baseline airway obstruction has been associated with airway wall thickening and asthma severity (22). Similarly, in guinea pigs, baseline obstruction has been linked to the degree of fibrosis, airway hyperreactivity, and antigen-induced airway obstruction in asthma models (15, 23). In the present study, baseline obstruction was indeed associated with the extent of fibrosis; however, it was not related to airway hyperreactivity or to the magnitude of antigen-induced airway obstruction. These findings suggest that increased baseline obstruction does not directly influence the mechanisms responsible for acute airway narrowing.

Previous studies from our group have shown that fibrosis and baseline airway obstruction progress gradually with repeated antigenic challenges. In contrast, neither airway obstruction nor hyperreactivity increase or decrease with successive challenges in the asthma model; instead, both responses remain remarkably stable from the first to the 12th antigen exposure (10, 15, 24).

Notably, reversal of airway fibrosis in guinea pigs using polymerized type I collagen did not reduce baseline airway obstruction, although it significantly decreased airway hyperreactivity. This finding indicates that subepithelial fibrosis, which contributes to airway wall thickening in this species, is not a determining factor in the increase of baseline obstruction (24). Therefore, other mechanisms involving different structural components of the airway may be responsible for limiting airflow under basal conditions.

Consistent with this interpretation, persistent airflow obstruction in patients with severe asthma has been associated with increased airway smooth muscle mass rather than submucosal fibrosis (25). Although airway smooth muscle mass did not change in the guinea pigs included in the present study, the observed increase in baseline obstruction likely reflects airway remodeling induced by repeated antigenic challenges. This remodeling may involve factors such as airway edema, mucus hypersecretion, or other processes; however, further studies are required to elucidate the precise mechanisms underlying this phenomenon.

Integrins are receptors involved in adhesion to the extracellular matrix and cellular adhesins. The β1 integrin subunit forms a non-covalently bound dimer with an α subunit (26). Research has shown that the β1 integrin subunit is naturally secreted and deposited in the guinea pig airway wall without changes during asthma (27). However, deposition of α1β1 and α2β1 integrins generated by shedding has been linked to fibrosis in the guinea pig asthma model (15). It is possible that the mechanism of shedding or secretion that induces the deposition of the β1 integrin subunit in the subepithelial region in NR guinea pigs is more pronounced than that observed in the asthma model. In human asthma, exacerbation leads to the presence of soluble integrins, particularly the β2 subunit, most likely due to matrix metalloproteinase-9-induced proteolytic cleavage (28). Evaluating the mechanism that produces high levels of the β1 integrin subunit deposition in the NR subepithelial area will be the subject of future studies.

With regard to airway smooth muscle, a recent study indicated that the smooth muscle of the NR phenotype exhibits high expression of laminin β2, suggesting that laminin plays a role in mitigating allergic responses and hyperreactivity (11). It has been noted that mediators such as IL-13 and IL-17, which are present in asthma, are associated with increased smooth muscle contractility through the NF-kB/Rho kinase/PIP5K1γ pathway and increased activity of the β1 integrin subunit (17). Considering the increased expression of this integrin observed in the asthma model, it is likely that the overexpression and activity of the β1 integrin subunit could be related to the development of hyperreactivity and contraction in airway smooth muscle.

Barometric plethysmography remains a controversial technique for measuring pulmonary function. However, our research has demonstrated that intravenous administration of acetylcholine or histamine induces a dose-dependent increase in Bi values in non-anesthetized guinea pigs, which correlates with total lung resistance (RL) values observed in separate groups of anesthetized animals. This suggests that Bi is a useful indirect measure of airway obstruction (20). In the present study, Bi was used to evaluate acute responses to OVA or histamine, revealing transient Bi increases in guinea pigs, which rapidly returned to baseline. This indicates that airway smooth muscle contraction was the primary response. Despite its limitations, Bi has been validated as a very sensitive indicator of increased specific airway resistance (29). Although our study did not include invasive validation methods, the existing literature supports the reliability of Bi in evaluating airway function. We acknowledge this limitation and suggest that future studies incorporate direct invasive measurements to further validate Bi.

A limitation of this study is its primary focus on histopathological and molecular features, without delving into the underlying mechanisms responsible for the differences between the asthma and NR phenotypes, and without conducting functional manipulations or mechanistic validations. The chronic antigen exposure protocol may not fully replicate all aspects of human asthma, which could affect the translational relevance of the findings. Furthermore, this study focused solely on male guinea pigs. This decision was based on previous observations indicating that sex hormones alter hormone receptor expression in rat airway smooth muscle (30) and that 17β-estradiol facilitates the relaxation of human airway smooth muscle by reducing intracellular Ca^2+^ levels and activating protein kinase A (31). Moreover, the estrous cycle in female guinea pigs, which lasts about 16 days, introduces cyclic variations that might affect airway smooth muscle contractility, though this aspect has not been fully studied. Consequently, the outcomes of our study should not be generalized across sexes. We acknowledge this limitation and emphasize that our findings cannot be directly applied to female guinea pigs. Future research should include sex-based comparisons to provide a comprehensive understanding of airway function across different sexes. Finally, due to the limited sample size, the analyses presented should be interpreted as exploratory and further studies with larger cohorts are needed to validate these findings.

This study provides translational relevance by modeling the heterogeneity of allergic airway responses observed in human asthma. The presence of asthma model and NR phenotypes despite antigen sensitization reflects clinical variability, where not all sensitized individuals develop functional asthma (32, 33). Baseline airway obstruction associated with subepithelial fibrosis and β1 integrin expression was dissociated from airway hyperresponsiveness, indicating remodeling-related airflow limitation, whereas increased β1 integrin expression in airway smooth muscle was specific to the asthma phenotype and associated with functional severity. Together, these findings highlight the usefulness of this model for distinguishing structural remodeling from functional impairment and for evaluating mechanisms related to asthma severity and prognosis.

In conclusion, the NR phenotype demonstrates significant development of subepithelial fibrosis accompanied by substantial deposition of the β1 integrin subunit in this region. Conversely, the expression of the β1 integrin subunit may play a role in the contraction and hyperreactivity of airway smooth muscle in the asthma model.

## Data Availability

The raw data supporting the conclusions of this article will be made available by the authors, without undue reservation.
